# Brazilian Protocol for Sexually Transmitted Infections 2020: acquired syphilis

**DOI:** 10.1590/0037-8682-616-2020

**Published:** 2021-05-17

**Authors:** Francisca Lidiane Sampaio Freitas, Adele Schwartz Benzaken, Mauro Romero Leal de Passos, Ivo Castelo Branco Coelho, Angélica Espinosa Miranda

**Affiliations:** 1 Ministério da Saúde, Secretaria de Vigilância em Saúde, Brasilia, DF, Brasil.; 2 Universidade de Brasília, Programa de Pós-Graduação em Saúde Coletiva, Brasília, DF, Brasil.; 3 Fundação de Medicina Tropical Doutor Heitor Vieira Dourado, Manaus, AM, Brasil.; 4 AIDS Healthcare Foundation, Los Angeles, Califórnia, USA.; 5 Universidade Federal Fluminense, Centro de Ciências Médicas, Niterói, RJ, Brasil.; 6 Universidade Federal do Ceará, Faculdade de Medicina, Fortaleza, CE, Brasil.; 7 Universidade Federal do Espírito Santo, Vitória, Brasil.

**Keywords:** Syphilis, Clinical protocols, Diagnosis, Therapeutics

## Abstract

The Clinical Protocol and Therapeutic Guidelines for Comprehensive Care of People with Sexually Transmitted Infections, published by the Brazilian Ministry of Health in 2020, includes updates concerning acquired syphilis. The document comprises rapid test use, safety and efficacy of benzathine benzylpenicillin, case follow-up, neurosyphilis clinical and laboratory management, approaching sex partners, assistance and monitoring of diagnosed pregnant women, and syphilis and HIV co-infection specificities, as well as a case notification summary. Health managers and professionals must be continuously trained so as to integrate care and surveillance, to strengthen actions for efficient control of syphilis, to broaden the search for sex partners, and to expand access of most vulnerable populations to health services.

## Highlighted excerpt

Most people with syphilis are asymptomatic; this contributes to the maintenance of the transmission chain. Without adequate treatment of pregnant women with syphilis, severe consequences can occur, such as miscarriage, prematurity, low birth weight, natimortality, and congenital syphilis.

## INTRODUCTION

This article summarizes the chapter on acquired syphilis, part of the Clinical Protocol and Therapeutic Guidelines (PDCT) for Comprehensive Care of People with Sexually Transmitted Infections (STI). The PDCT was approved by the National Committee for the Incorporation of Technologies to the Brazilian National Health System (Conitec), through Ordinance no. 42, of October 5, 2018^1^. When writing the Protocol, evidence available in literature for analysis was selected, and discussion among specialists was carried out. The PDCT was updated by the technical group and published in 2020 by the Health Surveillance Department of the Brazilian Ministry of Health. 

## EPIDEMIOLOGICAL ASPECTS

Syphilis is an STI caused by the *Treponema pallidum* bacteria, subspecies *pallidum*. Transmission is mainly sexual (oral, vaginal, or anal). It can also be transmitted vertically, with a fetal mortality rate higher than 40%^2^. 

Most people with syphilis are asymptomatic, which contributes to maintenance of the transmission chain. If the disease is not treated, it can lead to severe systemic complications after many years from the initial infection^3^ ^-^ ^5^. Without adequate treatment of pregnant women with syphilis, severe consequences can occur to the fetus or the conceptus, such as miscarriage, prematurity, low birth weight, natimortality, and early or late clinical congenital syphilis manifestations^6^.

*Treponema* penetrates the mucous membranes directly or enters through skin injuries. Transmission is higher at the infection’s early stages (primary and secondary syphilis), gradually decreasing over time^5^.

In 2016, the World Health Organization (WHO) estimated 6.3 million new cases of syphilis worldwide, with a 0.5% prevalence in men and women, and regional values varying from 0.1% to 1.6%^7^. In Brazil, a national study^8^ in 2016 showed a 0.6% syphilis prevalence in conscript young people, who were called for selection commissions, after the military enlistment stage. A high syphilis prevalence was observed in segments of critical populations in Brazil, such as men who have sex with men (9.9%)^9^, female sex workers (8.5%)^10^, and prisoners (3.8%)^11^.

The acquired syphilis detection rate increased from 59.1 cases per 100,000 inhabitants, in 2017, to 75.8 cases per 100,000 inhabitants, in 2018, with a higher increasing trend among the population between 20 and 29 years, from 2010 to 2018, according to data from the Notifiable Diseases Information System (Sinan)^12^.

## CLINICAL ASPECTS

To guide treatment and clinical and laboratory follow-up, syphilis infection is divided into the stages of recent syphilis (primary, secondary, and recent latent ones) with one year of evolution, and late syphilis (late latent and tertiary ones), longer than a year^4^.

**FIGURE 1:**
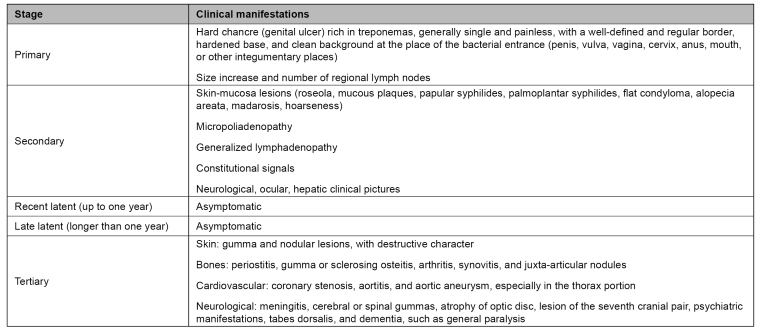
Acquired syphilis clinical manifestations and stages.

**Source:** Clinical Protocol and Therapeutic Guidelines for Comprehensive Care for People with Sexually Transmitted Infections, 2020^13^.

Figure 1 shows the clinical stages of acquired syphilis. At initial phases, symptomatology can vary and disappear, regardless of treatment. Clinical manifestations give room to clinical suspicion, but there is no exclusive signal or symptom; this can lead to misunderstanding concerning other pathologies and make diagnosis more difficult^13^.

Central nervous system (CNS) involvement can take place at any stage of clinical syphilis^14^. Early neurosyphilis manifests immediately after syphilis infection and meningitis and abnormalities in the cranial nerves^15^ ^,^ ^16^. Due to the era of antibiotics and the dominant use of beta-lactam antibiotics, neurosyphilis' clinical presentation has changed, with oligosymptomatic and atypical clinical pictures of the disease presenting increase^15^.

## DIAGNOSIS

To diagnose syphilis, clinical data, results from diagnostic tests, previous investigation history, and investigation on recent risky sexual exposure must be combined^17^. Analysis of sexual history is relevant to diagnosis, and requires professional skills and confidentiality assurance^18^.

Direct examinations and immunological tests are also used for diagnosis of syphilis. Direct examinations are those in which *T. pallidum* research or detection in biological samples collected directly from primary and secondary lesions is performed^19^.

**FIGURE 2:**
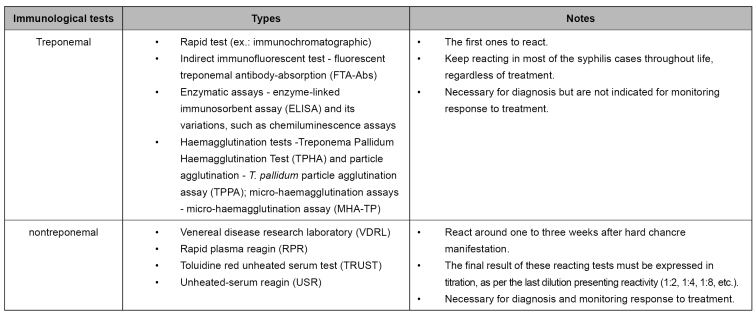
Immunological test for syphilis diagnosis.

**Source:** adapted from Clinical Protocol and Therapeutic Guidelines for Comprehensive Care for People with Sexually Transmitted Infections, 2020^13^.

Immunological tests (treponemal and nontreponemal) are the most common in clinical practice for tracking asymptomatic people and diagnosing symptomatic patients^5^. Their characteristic is the research of total antibodies in whole blood, serum, or plasma (Figure 2). Although there is a synthesis of specific IgM antibodies at the initial infection phase, such antibodies are also found in late infection stages; therefore, IgM tests are not recommended on their own^2^ ^,^ ^17^.

Treponemal tests detect specific antibodies produced against *T. pallidum* antigens. For example, rapid tests (immunochromatographic) can be highlighted, and they do not need a laboratory structure. In around 85% of cases, treponemal tests keep reacting throughout life (serological scar), regardless of treatment^19^, which allows for differentiating an active infection from a past one^5^.

Nontreponemal tests, such as the venereal disease research laboratory (VDRL), detect anticardiolipin antibodies nonspecific for *T. pallidum* antigens^19^. They are semi quantitative tests, as, in case of reacting results, dilution of the sample for titration of these antibodies is performed^2^. Such titration may vary, depending on the disease stage and on treatment or lack of it. Low titration (<1:4) of nontreponemal antibodies may be found in the infection’s early and late phases, persisting for months or years. For this reason, there is no specific cut-point, and any titration must be investigated as syphilis^13^. Non-negativity of nontreponemal tests after treatment is called a serological scar. This event can be temporary or persistent, and it can present low or high titration, depending on the initial titration found at the moment of diagnosis^4^ ^,^ ^17^.

**FIGURE 3:**
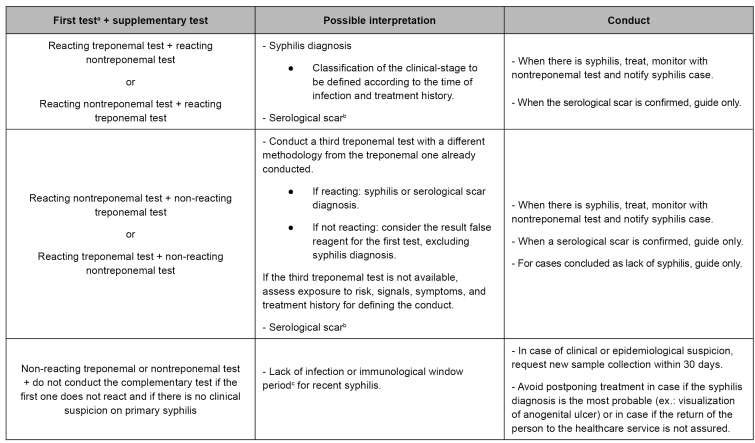
Treponemal and nontreponemal test results, interpretation, and conduct.

**Source:** adapted from the Clinical Protocol and Therapeutic Guidelines for Comprehensive Care for People with Sexually Transmitted Infections, 2020^13^.

**Notes:** a) Immediate treatment with benzathine benzylpenicillin after only one syphilis reacting test (treponemal or nontreponemal test) is indicated in situations described in item "Treatment", without exclusion of need for a second test for diagnosis definition; b) Documented previous treatment with titration drop in at least two dilutions; c) Immunological window: the period between infection and sufficient antibody production for immunological test detection.

Starting the investigation with a treponemal test, preferably the rapid test, is recommended due to its higher sensitivity^2^ ^,^ ^17^. With the results, there are various combinations of treponemal and nontreponemal tests’ use, with possible interpretation and respective conducts (Figure 3)^13^ ^,^ ^17^.

Healthcare professionals, especially medical and nursing, must explain the purpose of immunological tests in the request form for the laboratory network. In the diagnostic approach, three different situations are considered: syphilis diagnosis, when there is no rapid test in the healthcare service; syphilis diagnosis, after reacting rapid test at the place of service; and treatment follow-up, when the diagnosis and treatment have taken place, and it is needed to monitor the nontreponemal antibody titration for cure control, preferably with the same method used for diagnosis^13^.

There is no gold standard test for neurosyphilis diagnosis. It is based on a combination of clinical findings, alterations in the cerebrospinal fluid (CSF), and results of nontreponemal testing in CSF. It is hard to find patients with neurosyphilis who do not present pleocytosis^20^. Although levels of protein in CSF are not sensitive and specific to neurosyphilis diagnosis, protein standardization is essential for post-treatment monitoring^21^ ^-^ ^24^.

## TREATMENT

Immediate treatment is recommended, with benzathine benzylpenicillin, following a reacting - treponemal or nontreponemal - test for syphilis in the following cases, regardless of the presence of signs and symptoms:^13^ pregnant women; sexual violence victims; people with a chance of follow-up loss (those who will not return to the service); people with signs and symptoms of primary or secondary syphilis; and people without previous syphilis diagnosis.

**FIGURE 4:**
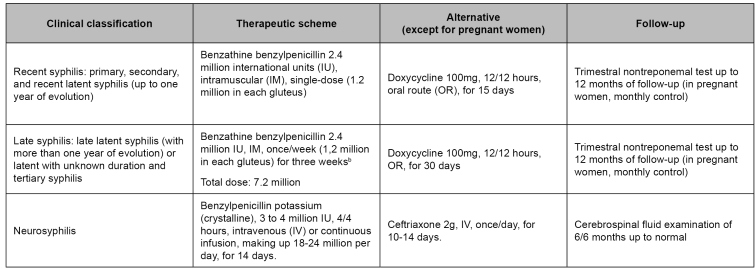
Treatment and follow-up of syphilis and neurosyphilis cases.

**Source:** adapted from Clinical Protocol and Therapeutic Guidelines for Comprehensive Care for People with Sexually Transmitted Infections, 2020^13^.

**Notes:** a) Benzathine benzylpenicillin is the only safe and efficient option for adequate treatment of pregnant women. Any other treatment conducted during pregnancy, for purposes of defining the case and therapeutic approach of congenital syphilis, is deemed inadequate treatment for the mother; consequently, the newborn will be notified as having congenital syphilis undergo clinical and laboratory assessment; b) The interval between doses must not be longer than 14 days. In such a case, the scheme must restart^19^.

After the first reacting test, treatment does not exclude the need for a second test, clinical and laboratory follow-up, and sex partners’ diagnosis and treatment. There are specific therapeutic schemes as per the clinical classification of syphilis (Figure 4)^13^. Solving signs and symptoms after treatment indicates a response to the therapy. A post-treatment follow-up must be conducted after the nontreponemal test for determining the adequate immune response^25^.

In order to guarantee availability of benzathine benzylpenicillin, it began to be acquired in a centralized way by the Brazilian Ministry of Health, as a strategic component of the pharmaceutical care in the Essential Medicines National List, from 2017^26^. Notified syphilis cases (acquired and in pregnant women) served as a base for calculating purchase and distribution^13^.

Benzathine benzylpenicillin must be administered through an intramuscular route (IM)^19^. The ventrogluteal region is the preferable place, as it is free from important vessels and nerves, and is a thinner subcutaneous tissue, which implies few adverse effects and less local pain^27^. Thigh vastus lateralis and dorsogluteal regions are other options for application. When application through the IM route is unfeasible in the indicated places due to the presence of silicone (prosthesis or industrial liquid), alternative treatment through oral route is recommended (Figure 4)^13^.

Jarisch-Herxheimer reaction is an episode that can happen in the first 24 hours after starting treatment with penicillin, mainly in primary and secondary syphilis. Its manifestation exacerbates skin lesions with erythema, pain or pruritus, general discomfort, fever, headache, and arthralgia^28^. Antipyretics can be used for controlling symptoms, but there is no evidence of reaction prevention^4^. People must be warned of the possibility of such benign and self-limited events^29^, and, especially, of the distinction regarding allergy to penicillin^30^.

According to a systematic review with meta-analysis, anaphylaxis risk in using benzathine benzylpenicillin was 0.002%, with the expectation of 0 to 3 cases of anaphylaxis per 100,000 treated patients of 13 studies. There was no anaphylactic reaction or death in the pregnant population due to the use of benzathine benzylpenicillin in 1,244 women, with a reported case of cutaneous rash^31^.

Adrenalin is the medicine chosen for anaphylactic reaction treatment. In this case, primary health care protocol recommendations regarding spontaneous demands and urgency and emergency must be followed^32^.

Healthcare professionals’ fear of adverse reactions arising from penicillin, beyond sporadic anaphylactic reactions, contributes to the loss of the opportune moment for the treatment of people with syphilis; also, it maintains the infection transmission chain and the occurrence of congenital syphilis^13^. Nursing professionals are backed by the Federal Nursing Council on extensive administration of benzathine benzylpenicillin in primary health care^33^ ^,^ ^34^.

From 80% to 90% of self-reported penicillin allergies are expected to be incorrect; it is challenging to differentiate reactions and disease symptoms in most cases^35^. Some specific situations do not characterize allergies, such as gastrointestinal symptoms, headaches, pruritus, family history, and suspected reactions that occurred more than ten years ago. Clinical history must be detailed for risk stratification of allergy to penicillin and to obtain adequate information and correct practice definition, preventing unnecessary referrals for desensitization in a hospital environment^36^.

Monitoring response to treatment is mandatory and must be performed throughout the outpatient healthcare network. The drop in immune response markers to *T. pallidum* evaluation uses as parameter the non-reacting nontreponemal test or the drop in titration in at least two dilutions within up to six months for recent syphilis and drop in titration in at least two dilutions within up to 12 months for late syphilis^4^ ^,^ ^37^ ^-^ ^40^.

The earlier the diagnosis and treatment, the faster the circulating antibodies will disappear, with negativity of nontreponemal tests or stabilizing this titration as low. The titration record of this nontreponemal examination at the beginning of treatment will serve as the base for clinical and laboratory monitoring^17^.

The following are criteria of benzathine benzylpenicillin retreatment:^13^ lack of titration reduction in two dilutions within six months (recent syphilis) or 12 months (late syphilis) after adequate treatment (ex.: from 1:32 to 1:8), or titration increase in two or more dilutions (ex.: from 1:16 to 1:64), or clinical signs’ and symptoms’ persistence or recurrence.

In reinfection cases, investigation of neurosyphilis through a lumbar puncture in the general population is recommended when there is no risk of sexual exposure. For people living with HIV, an investigation is recommended in all retreatment cases, regardless of occurrence or not of new exposure. After adequate treatment, when a new risk of sexual exposure during the analyzed period is dismissed, the persistence of reacting results in nontreponemal tests, with previous titration drop in at least two dilutions, is called a serological scar, and does not characterize therapeutic failure^13^.

For neurosyphilis, all people presenting reacting VDRL in CSF must be treated, regardless of neurological or eye signals, and of symptoms. Those presenting non-reacting VDRL in CSF and biochemical alterations in CSF and presence of neurological or eye symptoms or changes in CNS image characteristic for the disease, since other diseases cannot explain such changes, must also be treated. Patients with an initial negative examination in CSF must undergo control fluid puncture as well after six months from the end of treatment (Figure 4)^13^.

In the case of CSF alteration persistence, retreatment with benzathine benzylpenicillin is recommended. In blood samples, the drop in nontreponemal test titration in at least two dilutions or seroreversion for non-reagent may be a parameter to be considered as a response to neurosyphilis, especially in an unavailable lumbar puncture scenario^41^.

## SURVEILLANCE, PREVENTION, AND CONTROL

Acquired syphilis has been under compulsory notification in Brazil since 2010, as per Consolidation Ordinance no. 4, of September 28, 2017^42^. Such notification is mandatory for physicians, other healthcare professionals, or those responsible for public and private healthcare services providing care to patients^43^. Thus, the need for timely notification of all cases to Sinan is reinforced to subsidize the formulation and implementation of public STI policies in Brazil.

Between 46% and 60% of the sex partners of people with syphilis (primary and secondary) are estimated to be infected^44^. If there is recent exposure (up to 90 days), even in case the person presents non-reacting immunological tests^4^, the recommendation is the presumptive treatment with a single dose of benzathine benzylpenicillin 2.4 million international units (IU), IM (1.2 million IU in each gluteus). It must be stressed that the clinical assessment and laboratory follow-up are crucial^13^. The approach to the sex partners contributes to decreasing the infection burden in the community, tracking asymptomatic people, and identifying sexual risk networks^45^.

For clinical and laboratory follow-up of people with acquired syphilis, nontreponemal test titration must be conducted every three months up to the 12^th^ follow-up month (3, 6, 9, and 12 months). This monitoring contributes to classifying the response to treatment, identifying possible reinfection, and establishing adequate conduct for each case^13^.

In most laboratory routines, nontreponemal tests are not automatized, which can cause a difference between readings when different methods are used or performed by more than one observer. Thus, variations in dilution titration (ex.: from 1:16 for 1:8) do not have clinical significance. Follow-up is recommended, as possible, using the same diagnostic method^17^.

In the PDCT chapter on acquired syphilis, there is a section on the clinical decision algorithm for syphilis in pregnant women management of (acquired and in pregnant women), with a recommendation summary for case screening, diagnosis, treatment, notification and clinical and laboratory monitoring^13^.

## SPECIAL POPULATIONS AND SITUATIONS

### Pregnant women

Pregnant women must be tested for syphilis at their first prenatal care appointment (ideally in the first three months), at the beginning of the third trimester, and at the hospitalization for giving birth, in case of miscarriage or natimortality or exposure to risk or sexual violence history. Clinical and laboratory monitoring with nontreponemal testing must be conducted monthly during pregnancy^46^. After birth, this follow-up is trimestral up to the 12^th^ follow-up month^13^.

It is crucial to assure pregnant women and sex partners’ diagnosis and treatment and register the procedure on the prenatal care notebook. Such conducts contribute to avoiding that the newborn undergoes unnecessary biomedical interventions^46^. It is also essential to stimulate the father’s or partner’s participation throughout the prenatal care process for strengthening healthy affection relations^47^.

### HIV infections

For all people living with HIV diagnosed with syphilis, appointments with specialists must be early in case of neurological or eye signals or symptoms, and lumbar puncture is a diagnostic imposition. Recommendations for lumbar puncture in people living with HIV to investigate neurosyphilis encompass the presence of neurological or ophthalmologic symptoms, evidence of active tertiary syphilis, and clinical treatment failure, regardless of sexual history^13^.

In HIV infections, syphilis clinical manifestations and therapeutic response can be distinct due to each person's immunity. The presence of multiple chancres, higher frequency of secondary lesions, and Jarisch-Herxheimer reaction can be highlighted^48^ ^,^ ^49^. Diagnosis criteria and treatment of syphilis for people living with HIV are the same for people without HIV infection^4^.

The update of the PCTD chapter on acquired syphilis converges with the need to train health managers and professionals continuously, to integrate care and surveillance, strengthen effective syphilis prevention actions, tracking asymptomatic people, and case diagnosis, treatment, follow-up, and surveillance, in addition to broadening the search for sex partners, and expanding the access of the most vulnerable populations to the health services. 
